# Small molecule FAK activator promotes human intestinal epithelial monolayer wound closure and mouse ulcer healing

**DOI:** 10.1038/s41598-019-51183-z

**Published:** 2019-10-11

**Authors:** Qinggang Wang, Shyam K. More, Emilie E. Vomhof-DeKrey, Mikhail Y. Golovko, Marc D. Basson

**Affiliations:** 1 Department of Surgery, University of North Dakota School of Medicine & Health Sciences, Grand Forks, USA; 2 Department of Biomedical Sciences, University of North Dakota School of Medicine & Health Sciences, Grand Forks, USA; 3 Department of Pathology, University of North Dakota School of Medicine & Health Sciences, Grand Forks, USA

**Keywords:** Gastroenterology, Ulcers

## Abstract

GI mucosal healing requires epithelial sheet migration. The non-receptor tyrosine kinase focal adhesion kinase (FAK) stimulates epithelial motility. A virtual screen identified the small drug-like FAK mimic ZINC40099027, which activates FAK. We assessed whether ZINC40099027 promotes FAK-Tyr-397 phosphorylation and wound healing in Caco-2 monolayers and two mouse intestinal injury models. Murine small bowel ulcers were generated by topical serosal acetic acid or subcutaneous indomethacin in C57BL/6J mice. One day later, we began treatment with ZINC40099027 or DMSO, staining the mucosa for phosphorylated FAK and Ki-67 and measuring mucosal ulcer area, serum creatinine, ALT, and body weight at day 4. ZINC40099027 (10–1000 nM) dose-dependently activated FAK phosphorylation, without activating Pyk2-Tyr-402 or Src-Tyr-419. ZINC40099027 did not stimulate proliferation, and stimulated wound closure independently of proliferation. The FAK inhibitor PF-573228 prevented ZINC40099027-stimulated wound closure. In both mouse ulcer models, ZINC40099027accelerated mucosal wound healing. FAK phosphorylation was increased in jejunal epithelium at the ulcer edge, and Ki-67 staining was unchanged in jejunal mucosa. ZINC40099027 serum concentration at sacrifice resembled the effective concentration *in vitro*. Weight, creatinine and ALT did not differ between groups. Small molecule FAK activators can specifically promote epithelial restitution and mucosal healing and may be useful to treat gut mucosal injury.

## Introduction

Failure of mucosal healing is central to diseases as diverse as inflammatory bowel disease (IBD), peptic ulcer, and necrotizing enterocolitis (NEC). Failure to heal substantially impairs quality of life in these diseases and may result in risky surgery or mortality. Approximately 1 million people in the US are afflicted with IBD^1^, and 51,200 died of IBD in the US alone in 2013^2^. GI mucosal healing represents an equilibrium between injurious agents and the migration and proliferation of epithelial cells at the wound edge. Antacids or antisecretory drugs for ulcers and anti-inflammatory agents for IBD attempt to minimize further injury. However, intact mucosa resists noxious stimuli more effectively than mucosa in which the mucosal barrier has been breached. Therefore, promoting mucosal healing by epithelial sheet migration is a potentially synergistic target. Surprisingly, despite substantial research into growth factor and cytokine effects, no therapeutic agents promote mucosal healing directly in treating IBD, peptic ulcer, NEC, or other gut mucosal lesions, with the possible exception of sucralfate, which some have hypothesized to bind luminal growth factors and plaster them across peptic ulcers^3^.

Focal adhesion kinase (FAK) is a key regulator of epithelial sheet migration. FAK activation is a convergent target for many growth factors^4^. Inhibiting FAK inhibits migration^5^. However, activated FAK is decreased in migrating intestinal epithelial cells *in vitro*^5^ and at the edge of human mucosal ulcers^6^, making FAK an attractive target to promote mucosal healing. While searching the ZINC database^7^ for small molecules that would mimic a key subdomain of the *N*-terminal FERM domain of FAK and therefore might competitively inhibit FAK-AKT binding, we serendipitously identified 2 small molecules, ZINC40099027 and ZINC25613745 that actually activate FAK at concentrations as low as 10 nM^8^. We sought to evaluate whether one of these molecules, ZINC4009027, would promote intestinal epithelial monolayer wound closure via sheet migration *in vitro*, the extent to which the effects are due to FAK activation and to the stimulation, and the potential efficacy of one of these molecules *in vivo*.

We initially studied signaling and monolayer wound closure *in vitro* in human Caco-2BBE cells. Although originally derived from a colon cancer, these cells are highly differentiated, form electrically and morphologically tight monolayers in culture, and are a common model for the study of intestinal epithelial sheet migration^9–14^. We administered a single dose to mice intraperitoneally and measured serum levels to estimate a relevant dosing interval and then measured the effects of three days of parenteral treatment in two different murine ulcer models – ischemic ulceration induced by topical serosal acetic acid^12,15–17^ and indomethacin-induced ulceration^18,19^. Our observations presented here suggest that such drug-like small molecules can promote intestinal epithelial restitution *in vitro* and in mucosal healing in mice, at least in part by activating FAK.

## Results

### ZINC40099027 activates FAK in migrating and suspended cells

**Figure 1:**
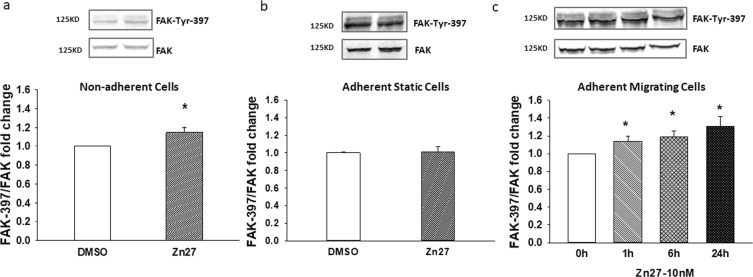
The effect of putative FAK activator ZINC40099027 on phosphorylation of FAK-Tyr-397 in human Caco-2 cells. Caco-2 cells were treated with 0.1%DMSO as a vehicle control or 10 nM ZINC40099027 (Zn27) for 1 hour. Total FAK served as loading control. (**a**) Shows representative blots and Tyr-397/FAK fold change in Caco-2 cells in suspension (n = 12, *p < 0.05). (**b**) Representative blots and Tyr-397/FAK fold change treated with ZINC40099027 in confluent adherent static Caco-2 cells. (n = 6). (**c**) Representative blots and Tyr-397/FAK fold change in migrating cells at various time points after treatment with 10 nM ZINC40099027(n = 7, *p < 0.05).

Treating suspended Caco-2 cells with 10 nM ZINC40099027 at 37 °C for 1 hour increased FAK-Tyr 397 phosphorylation vs. DMSO controls (Fig. 1a), by 14.8 ± 5% (n = 12, p < 0.05). This was consistent with our previous observations of ZINC40099027 in suspended SW620 cells^8^. Because intestinal epithelial cells adhere to the basement membrane and do not exist in suspension, we attempted to replicate this observation with ZINC40099027 in adherent cells. Surprisingly, 10 nM ZINC40099027 did not measurably activate FAK in static confluent Caco-2 cell monolayers on a collagen I matrix. (Fig. 1b). However, because we were interested in the potential effects of these compounds on epithelial sheet migration, we next evaluated the effect of ZINC40099027 in migrating Caco-2 cells, using a model of sparse seeding to create small islands of migrating cells as previously described^5^. Indeed, treating migrating Caco-2 cells with 10 nM ZINC40099027 increased FAK-Tyr 397 phosphorylation by 12.9 ± 5.7%, 19.1 ± 6.3%, 31.1 ± 10.6% at 1 hr, 6 hr, 24 hr respectively (Fig. 1c, n = 7, p < 0.05). These results demonstrate that ZINC40099027 activates FAK in both suspended and migrating Caco-2 cells.

### ZINC40099027 stimulates Caco-2 cell monolayer wound closure

**Figure 2:**
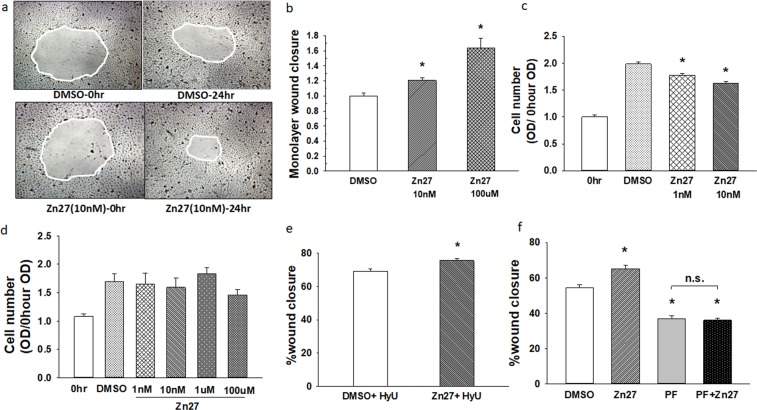
FAK activators stimulate Caco-2 cell monolayer wound closure. (**a**) Typical wound images treated with DMSO or ZINC40099027 at 10 nM. Images were taken at 0 and 24 hour time points. All images are 40x original magnification. (**b**) 10 nM or 100 µM ZINC40099027 accelerates wound closure in Caco-2 monolayers. (n = 32, pooled from 4 separate studies, *p < 0.05). (**c**) ZINC40099027 does not increase cell proliferation at 24 hours in migrating Caco-2 cells. (n = 24, pooled from 3 studies with similar results, *p < 0.05). (**d**) ZINC40099027 (1 nM–100 uM) does not affect cell number in Caco-2 confluent monolayers compared to control cells over 24 hour. (**e)** ZINC40099027 at 10 nM accelerates circular wound closure in Caco-2 monolayers on collagen I when proliferation is blocked by 4 mM hydroxyurea. (n = 28, pooled from 4 separate studies, *p < 0.05). (**f**) FAK inhibitor PF-573228 at 10 µM prevents ZINC40099027 stimulation of wound closure. (n = 16, *p < 0.01 compared to DMSO).

Incubation with 10 nM ZINC40099027 for 24 hours accelerated Caco-2 epithelial monolayer wound closure by 20.7 ± 3.9%, vs. wounded monolayers treated with vehicle alone. (Fig. 2a; n = 32, p < 0.05). To determine whether this effect would be more potent at a higher concentration, we treated wounded monolayers with 100 µM of ZINC40099027. Indeed, 100 µM ZINC40099027 accelerated wound closure by 63.1 ± 9.3%. (Fig. 2b; n = 12, p < 0.05).

Acceleration of wound closure could reflect increased proliferation or increased migration or both. To examine the effects of ZINC40099027 on proliferation, cell numbers were measured after incubation with ZINC40099027 for 24 hours. ZINC40099027 did not increase cell number in migrating cells at either 1 nM or 10 nM concentrations vs. DMSO-treated control cells (Fig. 2c) and showed no toxic decrease in cell numbers over 1 nM – 100 µM in confluent monolayers (Fig. 2d). We further investigated the ZINC40099027 effect on promoting wound closure using 4 mM hydroxyurea to prevent proliferation^20^. Even when proliferation was blocked by hydroxyurea, ZINC40099027 still accelerated wound closure in comparison to DMSO controls (Fig. 2e; n = 28, pooled from 4 separate studies, p < 0.05).

We next sought to determine whether ZINC40099027 stimulation of wound closure occurred because of FAK activation or some off-target effect. The FAK inhibitor PF-573228 (10 µM) reduced monolayer wound closure from 65.2 ± 1.8% to 36.7 ± 1.8% (n = 16, p < 0.01) in DMSO-vehicle-treated monolayers. Simultaneous treatment with PF-573228 and ZINC40099027 prevented the ZINC40099027 effect, yielding wound closure of 36.0 ± 1.1%, not different from wound closure after treatment with PF-573228 alone. (Fig. 2f). This suggested that ZINC40099027 stimulates migration via FAK activation.

### ZINC40099027 specificity to FAK activation in Caco-2 cells

**Figure 3:**
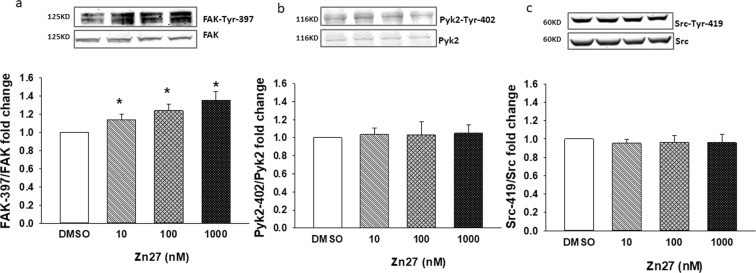
ZINC40099027 specificity for the stimulation of FAK phosphorylation in Caco-2 cells. (**a**) ZINC40099027 at 10–1000 nM dose-dependently stimulates phosphorylation of FAK at Tyr-397. (n = 6, *p < 0.05). (**b**) ZINC40099027 at 10–1000 nM does not stimulate phosphorylation of Pyk2 at Tyr-402. (n = 6), or (**c**) Src at Tyr-419 (n = 6).

To further pursue the specificity of ZINC40099027 activation of FAK, Caco-2 cells were treated with ZINC40099027 at 10–1000 nM for one hour. ZINC40099027 dose-dependently increased FAK-Tyr-397 phosphorylation, with a maximal 36.0 ± 9.7% increase at the highest concentration. All concentrations were statistically higher than DMSO controls (Fig. 3a; n = 6, p < 0.05). In contrast, even at 1000 nM, ZINC40099027 did not activate the close FAK paralog, proline-rich tyrosine kinase 2 (Pyk2), as measured by phosphorylation of Pyk2-Tyr-402^21–23^. ZINC40099027 also did not activate the non-receptor protein tyrosine kinase Src, which also localizes to the focal adhesion complex and is implicated in cell motility^21,24^, when we assessed Src-Tyr-419 phosphorylation. (Fig. 3b,c).

### ZINC40099027 promotes intestinal mucosal healing in an ischemic ulcer model

**Figure 4:**
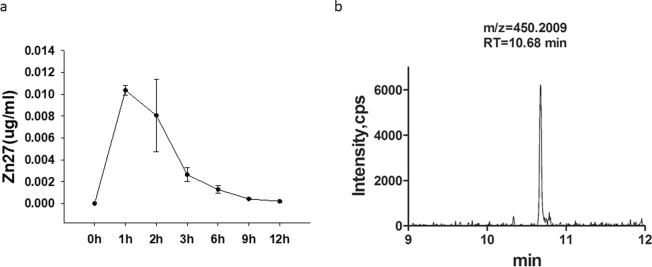
ZINC40099027 pharmacokinetics in mice. (**a**) Serum levels of ZINC40099027 are highest one hour after intraperitoneal injection of 900 µg/kg body weight, diluted in 0.9% saline to make 100 µl volume (n = 4). Serum ZINC40099027 levels then reduce time-dependently and rapidly. (**b**) Representative UPLC chromatogram analyzing serum ZINC40099027 concentration 6 hours after IP injection.

Prompted by these *in vitro* results, we studied the effect of ZINC40099027 on intestinal epithelial wound closure *in vivo*. To determine an appropriate dosage and administration interval, we first administered 900 µg/kg ZINC40099027 intraperitoneally to C57BL/6J mice and measured serum ZINC40099027 at various time points. Serum ZINC40099027 peaked one hour after intraperitoneal injection and then time-dependently decayed to baseline by 6 hours (Fig. 4a). Therefore, 900 µg/kg ZINC40099027 was administered intraperitoneally every 6 hours for 3 days in subsequent *in vivo* studies. Serum trough concentration levels of ZINC40099027 at 6 hours after the final dose in those studies was 2.07 ± 0.5 ng/ml (4.6 nM) (Fig. 4b), similar in magnitude although slightly lower than the 10 nM that was effective *in vitro*.

**Figure 5:**
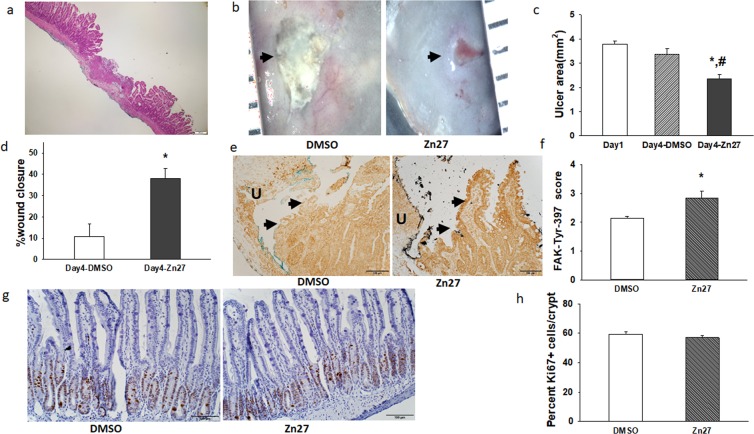
ZINC40099027 promotes intestinal mucosal healing in a murine ischemic ulcer model. (**a**) Hematoxylin and eosin staining of an ischemic ulcer at day 4. (**b**) representative images of jejunal ischemic ulcers in DMSO-treated mice and ZINC40099027-treated mice at day 4. (**c**) Ulcer area in mice receiving DMSO or ZINC40099027 at day 4 is compared to ulcer area in a group of mice sacrificed at day 1 before treatment with DMSO or ZINC40099027. ZINC40099027 substantially reduces ulcer area after 3 days of intraperitoneal injection every 6 hours. (n = 12, *p < 0.05 vs day1, # p < 0.05 vs DMSO). (**d**) Percentage of mucosal wound healing at day 4 in mice treated with DMSO or ZINC40099027 compared to the ulcer size at day 1 emphasizes the acceleration of ulcer healing by ZINC40099027. (n = 12, *p < 0.05). (**e**) Representative immunohistochemical images of FAK-397 phosphorylation in epithelium at the edge of the ulcer bed from DMSO control or ZINC40099027-treated mice. Arrows indicate epithelium at the edge of the ulcer. Dark brown staining indicates FAK-397 immunoreactivity. Green and black ink was used for coding sections. (**f**) Blinded immunoreactivity scores are increased in ZINC40099027-treated ulcers vs. DMSO-treated ulcers (n = 5, *p < 0.05). **(g**) Representative hematoxylin –counterstained immunohistochemical images of Ki67-stained crypts from DMSO or ZINC40099027-treated mice. Dark brown staining indicates Ki-67 immunoreactivity. (**h**) The percentage of Ki67-positive cells per small intestinal crypt did not differ between in DMSO or ZINC40099027-dosed mice.

We first studied ZINC40099027 effects in an acetic-acid-induced ischemic ulcer model described previously^16^, in which reproducible circumscribed ischemic ulcers are created within the small bowel by topical application of an acetic-acid-soaked disk of filter paper to the serosa of the bowel, without opening it. We sacrificed a group of mice after 24 hours to measure baseline ulcer size, and then treated the remaining mice with either 900 µg/kg ZINC40099027 or the DMSO vehicle every 6 hours intraperitoneally. Typical jejunal ulcer histology at day 4 is shown in Fig. 5a. Mice receiving ZINC40099027 for three days exhibited smaller ulcers than those receiving the DMSO control (Fig. 5b,c; 2.35 ± 0.18 vs. 3.38 ± 0.22 mm², n = 12, *p* < 0.05). To evaluate the wound healing rate, we compared the average day 4 ulcer size to that at day 1. The percentage of wound closure was markedly higher in ZINC40099027-dosed mice (Fig. 5d; 37.9 ± 4.8% vs 10.8 ± 5.7%, *p* < 0.01). As analyzing male and female mice separately suggested no gender effect, we pooled male and female mice for final analysis.

Blinded immunohistochemical assessment demonstrated higher immunoreactivity for phosphorylated FAK in the epithelium at the edge of ZINC40099027-treated ulcers vs. DMSO-treated ulcers (Fig. 5e,f; 2.83 ± 0.25 vs 2.15 ± 0.06 on a 1 to 4 scale, n = 6, *p* < 0.05). In contrast, we found no significant difference in the percentage of Ki67-positive cells per crypt between mice given ZINC40099027 or DMSO control (Fig. 5g,h; 59.45 ± 1.28% vs 56.93 ± 1.30%, n = 40).

### ZINC40099027 promotes indomethacin-induced small intestine injury mucosal healing

**Figure 6:**
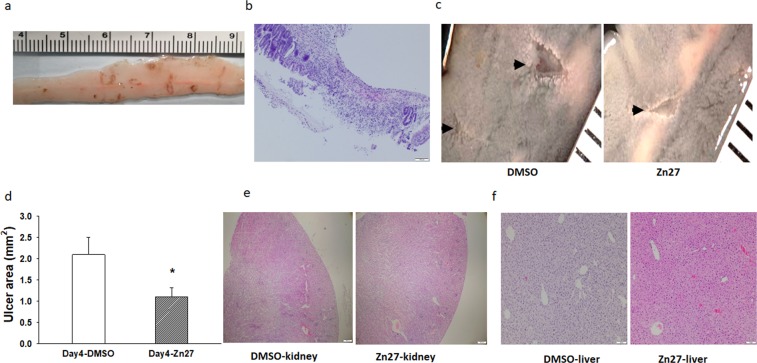
ZINC40099027 promotes the healing of indomethacin-induced small intestine injury in mice and does not affect murine kidney and liver morphology. (**a**) Typical macroscopic small intestinal ulcerative lesions induced by a single administration of indomethacin at a dose of 15 mg/kg subcutaneously at 24 hours after administration. (**b**) Hematoxylin and eosin staining of a typical indomethacin-induced small intestinal ulcer at day 4. **(c**) Representative images of ulcers from DMSO-treated mice and ZINC40099027-treated mice at day 4 suggest that ZINC40099027-treated mice have smaller ulcer areas than mice treated with only the DMSO vehicle. (**d**) Quantitation of total small intestinal ulcer area in DMSO-treated mice and ZINC40099027-treated mice demonstrates that ZINC40099027 reduces the total ulcer area after 3 days of intraperitoneal injection every 6 hours, (n = 22 in DMSO, n = 25 in ZINC40099027, *p < 0.05). (**e**) There is no obvious difference in renal or (**f**) liver morphology between typical hematoxylin and eosin stains of kidneys from mice treated with DMSO or ZINC40099027 for three days beginning one day after ulcer induction by subcutaneous injection of 15 mg/kg indomethacin, (50× magnification kidney, 100× magnification liver).

In a second *in vivo* mouse model, 8–10 week non-fasted C57BL/6J mice were given 15 mg/kg indomethacin subcutaneously, followed one day later by intraperitoneal DMSO or ZINC40099027 injections every six hours for the next three days. Typical indomethacin induced intestinal ulcerative lesions at 24 hours are shown in Fig. 6a. By day 4, multiple ulcerative lesions were seen throughout the small intestine. (Fig. 6b,c). By day 4, four mice had died in the DMSO group, and only one mouse had died in the ZINC40099027 group out of twenty-six mice per group. Among the surviving mice, ZINC40099027 treatment reduced the total ulcer area to 1.11 ± 0.40 mm² compared to 2.09 ± 0.21 mm² in the DMSO control (*p* < 0.05 Fig. 6d). This suggested that ZINC40099027 stimulated indomethacin-induced ulcer healing similarly to ischemic ulcers. Analyzing male and female mice separately suggested no gender effect, so we pooled them. Histological evaluation after hematoxylin and eosin staining revealed no obvious kidney or liver histopathology difference between the DMSO-treated and ZINC40099027-treated mice in the indomethacin model (Fig. 6e,f). The serum ZINC40099027 concentration at the end of the three days of treatment in this study was 1.81 ± 0.2 ng/ml six hours after the last dose at the time of sacrifice, approximately equivalent to 4.04 ± 0.58 nM (not shown).

### Three days of ZINC40099027 treatment does not affect murine serum creatinine or alanine aminotransferase

Although we did not perform formal toxicity studies, we did measure serum creatinine and alanine aminotransferase (ALT) levels as indicators of renal and hepatic function in both ulcer models. (Table 1). Serum creatinine remained within the normal range in all mice in each study, without any statistical difference between the treatment groups. In the ischemic ulcer model, the serum ALT level was higher in the mice receiving ZINC40099027 than in DMSO-treated mice, but all ALT levels were still within the normal range in these mice (7.63–53.1 U/L, MyBioSource’s reference range). In the indomethacin-induced ulcer model, ALT was higher than normal in both DMSO-treated mice and ZINC40099027-treated mice (77.1 ± 12.19 U/L and 69.1 ± 10.54 U/L respectively), but the ZINC40099027 mice did not show higher levels than those treated with the DMSO vehicle. Moreover, four days after mice received only a single subcutaneous ethanol injection at an amount equivalent to the ethanol vehicle used to dissolve the indomethacin, without any DMSO or ZINC40099027, ALT levels were 77.3 ± 2.04 U/L, similar to ALT levels in mice that had been treated with indomethacin dissolved in ethanol along with ZINC40099027 or its DMSO vehicle.

**Table 1:** Mouse serum creatinine and ALT after 1 day ulcerogenesis followed by DMSO or ZINC40099027 treatment for 3 days.

	Normal Range	Ischemic ulcer model		Ethanol only	Indomethacin model	
		DMSO (n = 5)	Zn27 (n = 5)		DMSO (n = 6)	Zn27 (n = 6)
Serum creatinine	0.06–16 mg/dl	0.109 ± 0.015	0.09 ± 0.019	**N.A**.	0.116 ± 0.022	0.068 ± 0.012
Serum ALT	7.63–53.1 U/L	11.44 ± 1.56	18.61 ± 3.01	77.3 ± 2.04	77.1 ± 12.19	69.1 ± 10.54

### ZINC40099027 does not increase weight loss in intestinal ulcer models

ZINC40099027 does not increase weight loss in intestinal ulcer models: We also examined weight loss as a possible indicator of systemic toxicity. Both DMSO-treated and ZINC40099027-treated mice lost weight in both the ischemic ulcer and indomethacin mouse models, likely reflecting the stress of these models. ZINC40099027-treated mice lost 7.67 ± 1.17% of their body weight in the ischemic ulcer model while DMSO-treated mice lost 9.65 ± 1.14%. Similarly, in the indomethacin model, ZINC40099027-treated mice lost 3.62 ± 0.88% of body weight compared to 4.79 ± 0.89% in DMSO-treated mice. Thus, ZINC40099027-treated mice did not exhibit more weight loss in these studies, and indeed tended to lose less body weight than DMSO controls, although this did not achieve statistical significance in either model.

## Discussion

Most therapy for diseases of gastrointestinal healing is directed at reducing or neutralizing the injurious agent(s). We treat peptic ulcers with antacids, antisecretory agents, and antibiotics to eliminate Helicobacter pylorii. We treat inflammatory bowel disease with agents to reduce inflammation. Such interventions are important and effective, but gastrointestinal mucosal healing represents an equilibrium between the negative effects of noxious stimuli and the positive effects of ongoing attempts at healing. We identified a small molecule that activates FAK *in vitro* in the Caco-2BBE colon cancer cell line. These results suggest that ZINC40099027 has drug-like properties that can stimulate FAK phosphorylation and promote the healing of intestinal epithelial wounds *in vitro* and *in vivo*. This more rapid wound closure is unlikely to reflect a mitogenic stimulus since *in vitro* cell numbers did not increase after treatment, monolayer wound closure was still accelerated in the presence of hydroxyurea to prevent cell proliferation, and Ki-67 staining did not change *in vivo*.

FAK is critical for epithelial cell motility^22,23,25^. However, FAK activation decreases at the edges of intestinal epithelial monolayer wounds *in vitro*^26^ and human gastric and intestinal ulcers *in vivo*^6^. FAK therefore seems an attractive target for pharmacotherapy to promote mucosal wound healing. ZINC40099027 seems to function by activating FAK since a FAK inhibitor prevented the stimulation of monolayer wound closure by ZINC40099027. Specificity is suggested by the observation that ZINC40099027 activated neither the most closely related tyrosine kinase Pyk2 nor the tyrosine kinase Src that colocalizes in the focal adhesion complex even at 100 times higher concentrations than those required for stimulation of FAK phosphorylation. Although we did not measure FAK activity directly, FAK autophosphorylation at tyrosine 397 represents the initial step in FAK phosphorylation and is frequently used as an indicator of FAK activation *in vitro* and *in vivo*^27–30^.

The effect of one hour of 10 nM ZINC40099027 treatment on FAK-Tyr-397 phosphorylation may seem modest. However, we and others have previously studied the significance of increases^31–35^ and decreases^36–38^ of similar magnitude in FAK phosphorylation in other contexts. Moreover, our results suggest that this small effect accelerates Caco-2 cell monolayer wound closure by 20%. At higher concentrations or longer times, we observed more substantial FAK activation or more rapid wound closure *in vitro*. For instance, at 100 µM, ZINC40099027 accelerated monolayer wound closure by 63%. Our *in vivo* observations clearly demonstrate smaller ulcers in ZINC40099027-treated mice in two different models, even with a trough 4.6 nM serum concentration, slightly lower than the 10 nM we found effective *in vitro*. Our *in vitro* observations of dose-dependency raise the possibility that a higher dose of such a molecule accelerate mucosal healing even more substantially. We observed only a slight decrease in cell number in migrating cells and no change in cell number in the confluent monolayers that might better model the intact unwounded mucosa in the rest of the gut, but this awaits further study and the possibility of GI or other toxicity at a higher dose. Our dosing here was conservative to avoid such potential toxicity.

Changes in FAK have previously been described in intestinal epithelial migration *in vitro*^26,39^, including at the leading edge during cell migration in monolayer wounds^40^. Reducing FAK by siRNA has been reported to inhibit cell migration *in vitro*^41^. The observation that FAK (Y397) immunoreactivity increased in epithelium at the ulcer edge of ulcer after ZINC40099027 treatment suggests that this small molecule promotes mucosal healing by increasing the activated FAK in migrating epithelial cells during restitution. The observation that ZINC40099027 actually inhibited Caco-2 cell proliferation slightly in the migrating Caco-2 cells and did not change intestinal epithelial proliferation *in vivo* suggests that this small molecule promotes mucosal healing by increasing migration rather than by stimulating proliferation, consistent with the parallel *in vitro* observation that the ZINC40099027 effect is maintained in the face of proliferative blockade by hydroxyurea.

We observed FAK activation in suspended or migrating Caco-2 cells but not in confluent static Caco-2 monolayers. We^5,42^ and others^43,44^ have previously described substantial differences in signaling networks between static and motile epithelial cells. We cannot tell whether FAK is simply not activated by ZINC40099027 in static cells or whether the activation is weak and lost behind background FAK activation by other stimuli. However, this observation suggests that therapeutic intervention by a small molecule targeted at this mechanism might have a greater effect on migrating gut epithelial cells at the edges of mucosal injury than on static epithelial cells elsewhere. Epithelia close wounds by sheet migration, while other cell types migrate individually^45–47^, so whether FAK is activated by these compounds in migrating cells of other types, such as endothelial or vascular smooth muscle cells, also awaits study. Although we did not perform formal toxicological studies, ZINC40099027 treatment did elicit obvious toxicity. All mice in both ulcer models lost weight, likely reflecting the effects of their procedures and/or ulcerations, but mice treated with ZINC40099027 tended to lose less weight in each model. Serum creatinine remained within the normal range in mice in each model. We did observe a slight elevation of ALT in mice treated with ZINC40099027 in the ischemic ulcer model, but ALT levels even in these mice remained within the normal range. In the indomethacin-ulcer model, ALT was elevated similarly in both vehicle-treated and ZINC40099027-treated mice. This was likely an effect of the alcohol used to deliver the indomethacin since mice treated with alcohol alone, without indomethacin, DMSO, or ZINC40099027, displayed a similar elevation. ALT did not differ between vehicle-treated and ZINC40099027-treated mice in the indomethacin model. Hepatic (and renal) morphology seemed grossly normal after treatment.

The mechanism by which these molecules activate FAK awaits further study. They were originally selected because we believed their conformation to resemble a subdomain of the *N*-terminal FERM domain of FAK^8^. FAK activation is traditionally believed to occur when the autoinhibitory FERM domain swings away from the rest of the molecule, allowing it to phosphorylate itself at Tyrosine 397^28^. More recently, a second model has been proposed in which FAK dimerizes, also via the FERM domain, and one molecule of FAK can activate another^48^. Thus, it is possible that these molecules could interact with FAK and modulate FAK-FAK interactions. However, they could also inhibit the interaction of some other molecule with FAK, such as a tyrosine phosphatase or an adapter protein that protects the molecule from tyrosine phosphatases. This awaits further study.

Although ZINC40099027 seems relatively compliant with the “rule of five”^49^, there is a large gap between such a theoretical structural analysis and development of a drug. Issues of pharmacokinetics, bioavailability, specificity and toxicity await further exploration. Nevertheless, this molecule promotes FAK activation even at relatively low nanomolar concentrations, and this activation does seem to stimulate wound closure without obvious short term toxicity.

Mucosal healing is a complex process requiring not only epithelial sheet migration but also epithelial proliferation, angiogenesis, and reconstitution of the basement membrane and submucosal tissues^50^. Not only epithelial cells, but also endothelial and vascular smooth muscle cells, fibroblasts, inflammatory other cell types are also involved, and these cells interact with each other^51–53^. Further exploration seems warranted to determine whether FAK activation by similar compounds can be adapted either alone or synergistically with agents that target these other processes into a therapeutic tool to promote gastrointestinal mucosal healing after injury.

## Methods

All methods were performed in accordance with relevant guidelines and regulations of the University of North Dakota.

### Materials

Dulbecco’s modified Eagle’s medium and Trypsin-EDTA were from Thermo Fisher (Waltham, MA). Secondary antibodies anti-rabbit 800, anti-mouse 680, anti-mouse 680 and anti-mouse 800 were from LI-COR (Lincoln, NE), FAK inhibitor PF-573228 from Selleck Chemicals (Houston, TX), indomethacin and hydroxyurea from Sigma Aldrich (St.Louis,MO) and glacial acetic acid from Fisher Chemical (Cat# A38S-500). Antibodies to FAK-Tyr-397(ab81298,1:1000 dilution), Pyk2-Tyr-402(ab4800,1:1000 dilution), Src(ab16885,1:1000 dilution), and Src-Tyr-419(ab185617,1:1000 dilution) were from Abcam (San Francisco, CA). We also used antibodies to total FAK (Anti-FAK, clone 4.47, 05–537, 1:1000 dilution EMD Millipore, Temecula, CA), and Pyk2 (3292 s, 1:1000 dilution, Cell Signaling technology Danvers, MA). ZINC40099027 was from Enamine (Monmouth Jct., NJ).

### Cell culture

Caco-2 cells from ATCC (Manassas, VA) were cultured as previously described^20^. To study cells in suspension, 80–90% confluent Caco-2 cells were seeded into cell culture plates pacificated with 1% heat inactivated bovine serum albumin to prevent adhesion and avoid adhesion-associated background FAK activation. These suspended cells were treated with DMSO (0.1%) or 10 nm ZINC40099027 for 1 hour before harvesting for Western blotting.

To study static or migrating cells, Caco-2 cells were seeded onto bacteriologic plastic dishes pre-coated with type I collagen (Sigma, St. Louis, MO) using ELISA coating buffer as described^34,54^. Static cells were studied within 1–2 days after confluence. Islands of migrating cells were created by seeding cells at 750 cells/cm² density into collagen I coated 100 × 20-mm bacterial petri dishes as described^5^. Cells were allowed to proliferate and migrate for 4 days, creating small islands of outwardly migrating cells. The cells were then treated with either DMSO (0.1%) or 10 nm ZINC40099027 before harvesting the cells for Western blot. We measured cell number using a Cell Counting Kit-8 (Dojindo, Japan) in static or migrating Caco-2 cells after 24 hour treatment with varying ZINC40099027 concentrations.

### Western blotting

Western blots were performed as described^55^ and detected by LICOR –Odyssey-Fc imaging (LI-COR Biosciences, Lincoln, NE). Densitometry was conducted on exposures within the linear range.

### Wound closure *in vitro*

Caco-2 cells were seeded at 80% confluence into collagen I coated 6-well plates. After 48–72 hours, when the cells had reached 100% confluence, wounds were made in the monolayer with non-barrier autoclaved 200 ul tips. Wound images were captured using an inverted light microscope (OLYMPUS CK2, Center Valley, PA) at 0 hours and 24 hours after treatment with DMSO or ZINC40099027. Wound areas were measured with Image J software.

### Animals

Eight to ten week old C57Bl/6J male and female mice from Jackson Laboratory (Bar Harbor, Maine) were bred and housed in temperature-controlled rooms with a 12-h light/12-h dark cycle at 22–24 °C and 50–60% humidity. All animals had free access to standard laboratory chow and tap water. All animal procedures were approved by the University of North Dakota Institutional Animal Care and Use Committee (protocol #1612–4, #1805-1).

### LC-MS analysis for serum concentrations

For initial pharmacokinetic studies, C57Bl/6J mice received a single intraperitoneal injection of 900 ug/kg dose of ZINC40099027. Mice were anesthetized with isoflurane. Blood was collected by cardiac puncture, transferred to a 1.5 ml eppendorf tube, left at room temperature for 30–60 min to form a clot, and centrifuged at 2000g for 10 minutes. Serum protein was precipitated from the supernatants using methanol 1:4 by volume. This was stored at −20 °C until assay of serum ZINC40099027 was assayed on a LC-MS Synapt G2-S Q-TOF mass spectrometer (Waters, Milford, MA) coupled to an ACUITY UPLC system with autosampler (Waters). MassLynx V4.1 software (Waters) was used for instrument control, acquisition, and sample analysis. For quantification, 450.2005 ion was extracted with a 0.02 ppm mass window and the peak area was calculated. Quantification was performed against a ZINC40099027 external standard using a generated response curve.

Mouse serum creatinine levels were determined using a Mouse Creatinine kit following manufacturer’s protocols (Crystal Chem, Elk Grove Village, IL, Cat No. #80350). Serum Alanine Aminotransferase (ALT) levels were assessed using a Mouse ALT ELISA kit following manufacturer’s protocols (MyBioSource, San Diego, CA, Cat No. MBS264717).

### Ischemic ulcer induction

Eight-ten week old C57BL/6J mice were anesthetized with isoflurane. Laparotomy exposed the jejunum. Ischemic jejunal ulcers were created by placing a 75% acetic acid-saturated circular filter disks (3.14 mm²) directly on the antimesenteric serosa for 15 seconds, without opening the bowel, as described^16^. This produces reproducible ischemic mucosal ulcers 24 hours later. Large blood vessels were avoided in disk placement. Some mice were sacrificed by cervical dislocation after isoflurane anesthesia at day 1 for baseline ulcer measurement. Animals were then randomly assigned to receive either intraperitoneal injection of ZINC40099027 at 900 ug/kg or DMSO vehicle control every six hours for three days after the first 24 hour period. At day 4, mice were anesthetized with isoflurane, blood was drawn by cardiac puncture for assay of serum levels of creatinine, ALT, and ZINC40099027, and animals were sacrificed by cervical dislocation before removing the jejunal ulcer segment. The resulting ulcers were imaged with an OLYMPUS Q Color 5 digital camera. Ulcer area was measured with Image J software. Area measurements were made by an observer blinded to animal assignment to each group. We compared ulcer sizes in mice sacrificed at day 4 after ulcer induction between the two groups (ZINC40099027 and DMSO vehicle) and to their day 1 counterparts who had received no drug.

### Indomethacin ulcer induction

To induce small intestinal lesions, non-fasted animals were given a single subcutaneous injection of indomethacin at a dose of 15 mg/kg dissolved in 100% ethanol at 100 ul. Animals were then randomly assigned to receive either intraperitoneal injection of ZINC40099027 at 900 ug/kg or DMSO vehicle control every six hours for three days after allowing 24 hours for ulcer induction. At day 4, mice were anesthetized with isoflurane, blood was drawn by cardiac puncture for assay of serum levels of creatinine, ALT, and ZINC40099027, and animals were sacrificed by cervical dislocation before removing the small bowel for measurement of ulcers. In addition, small bowel ulcer tissue, liver, and kidney were saved in some animals for histological examination. The full length of the small intestine was excised at day 4 at sacrifice. The small intestine was gently washed with phosphate buffered saline, fixed in neutral-buffered 10% formalin for 5 minutes, and then opened along the anti-mesenteric attachment for imaging. Mucosal ulcers were imaged with an OLYMPUS Q Color 5 digital camera. Ulcer area was measured with Image J software and we summed the total area of all ulcers within each animal’s small bowel to achieve a final total ulcer area. Area measurements were made by an observer blinded to animal group assignments.

### Histology

Mouse small intestine, kidney, and liver tissues were fixed in neutral-buffered 10% formalin solution for 48 hours, and then transferred into phosphate buffered saline at 4 °C for storage until processing as paraffin-embedded tissue blocks. Five micron sections stained with hematoxylin and eosin.

### Immunohistochemistry

Paraffin-embedded tissue sections were deparaffinized and subjected to antigen retrieval by boiling in sodium citrate (pH 6.0) to expose target proteins. Sections were incubated with 0.3% hydrogen peroxide for 30 minutes at room temperature and then blocked with 10% normal goat serum. Tissue samples were then incubated for 1 hour at room temperature with phospho-FAK(Tyr397) antibody (1:1000, Cat#700255, ThermoFisher) or anti-Ki-67 (1:200; Cat# ab16667, Abcam) antibody followed by incubation with a biotinylated anti-rabbit secondary antibody (Cat# PK-6101, Vector Laboratories, Inc.) and then incubation with avidin–biotin complex reagent (Cat# PK-6101, Vector Laboratories, Inc.) for 30 minutes at room temperature. Reactions were detected with 3,3′-diaminobenzidine solution (Cat#ab64238, Abcam). Some slides were counterstained with hematoxylin. Intensity of immunoreactivity for FAK phosphorylation in the migrating epithelium at the edge of the ulcer was scored by two blinded observers, with 0 being the least amount of detectable positive staining and 4 being the highest positive staining. Cellular proliferation in intestinal crypts was evaluated using Ki-67 immunolabeling. All of the epithelial cells in each crypt were counted and the percentage of cells with nuclear staining for Ki-67 was recorded. Assessment was independently performed by two authors in a blinded manner with similar results.

### Statistical analysis

Results were expressed as means ± standard error. Statistical analysis was performed using paired or unpaired t-tests or ANOVA or Chi-squared test as appropriate. A P-value of < 0.05 was considered statistically significant.

## Supplementary information

Supplementary information
