# Supplementary material for: Small molecule FAK activator promotes human intestinal epithelial monolayer wound closure and mouse ulcer healing
Source: Sci Rep. 2019 Oct 11;9:14669. doi: 10.1038/s41598-019-51183-z (PMC6789032; doi:10.1038/s41598-019-51183-z)

Title- *Small molecule FAK activator promotes human intestinal epithelial monolayer wound closure and mouse ulcer healing*

Authors-

Qinggang Wang[1], Shyam K. More[1], Emilie E. Vomhof-DeKrey[1], Mikhail Y. Golovko[2], Marc D. Basson[1,2,3]

1. Department of Surgery, University of North Dakota School of Medicine & Health Sciences

2. Department of Biomedical Sciences, University of North Dakota School of Medicine & Health Sciences

3. Department of Pathology, University of North Dakota School of Medicine & Health Sciences

Figure 1a

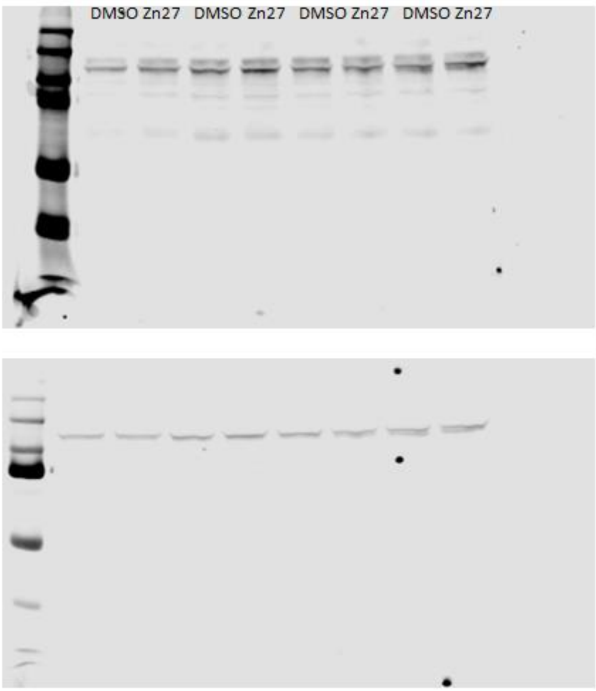

Figure 1b

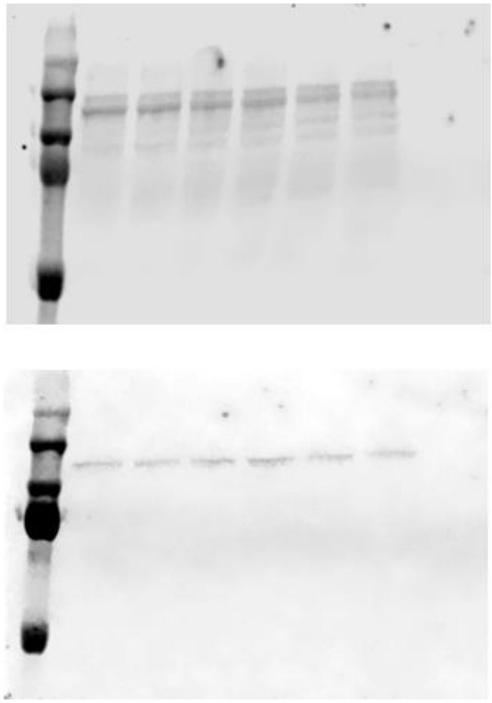

Figure 1c

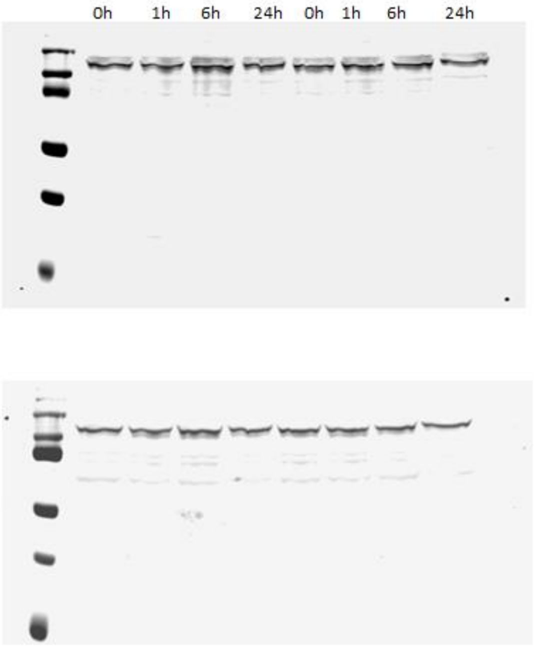

Figure 3a

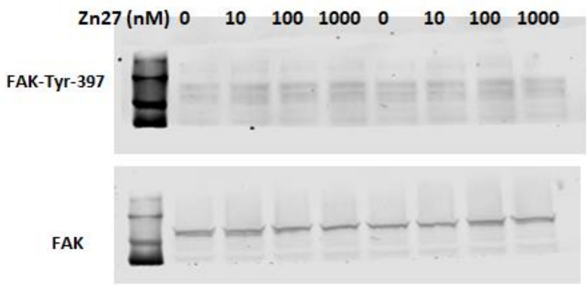

Figure 3b

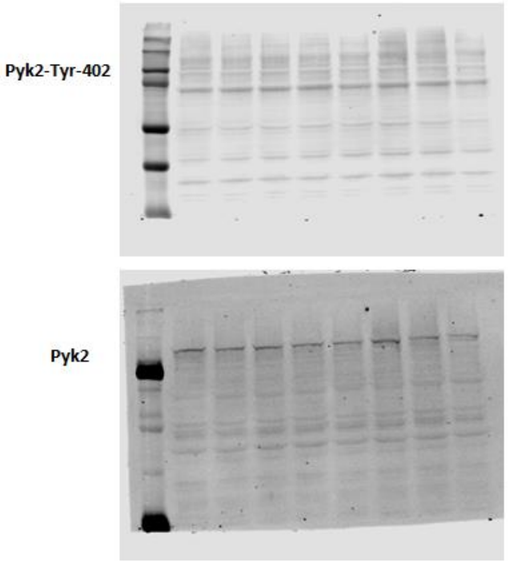

Figure 3c

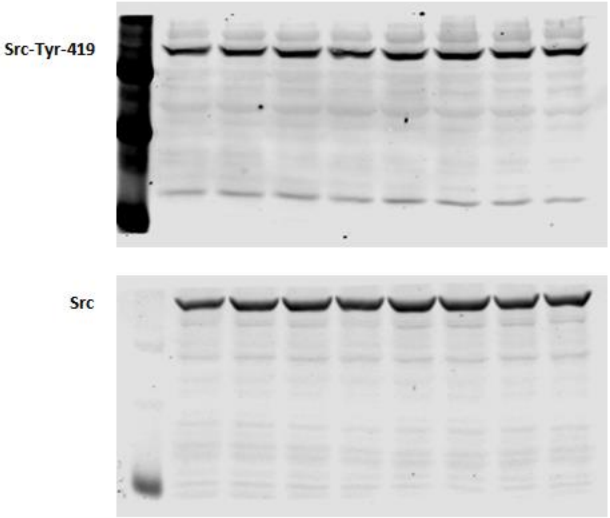

Supplement: Supplementary file 1 — Supplementary information [file 41598_2019_51183_MOESM1_ESM.pdf]
